# MiR‐139‐5p inhibits the tumorigenesis and progression of oral squamous carcinoma cells by targeting *HOXA9*

**DOI:** 10.1111/jcmm.13282

**Published:** 2017-08-05

**Authors:** Kai Wang, Jun Jin, Tengxiao Ma, Hongfeng Zhai

**Affiliations:** ^1^ Department of Plastic Surgery Henan Provincial People's Hospital Zhengzhou Henan China

**Keywords:** oral squamous cell carcinoma, miR‐139‐5p, *HOXA9*

## Abstract

Our study sought to clarify the effects of microRNA‐139‐5p (miR‐139‐5p) in the tumorigenesis and progression of oral squamous cell carcinoma (OSCC) by regulating *HOXA9*. MiR‐139‐5p and *HOXA9* expression in OSCC tissues, tumour adjacent tissues, OSCC cells and normal cells were tested by qRT‐PCR. SAS and CAL‐27 cell lines were selected in among four OSCC cell lines and then transfected with miR‐139‐5p mimics, pEGFP‐*HOXA9* and cotransfected with miR‐139‐5p mimics + pEGFP‐*HOXA9*. We used MTT, colony formation, transwell and wound healing assays to analyse cell viability, proliferation, invasion and migration. The target relationship between miR‐139‐5p and *HOXA9* was verified by luciferase reporter assay and Western blot, respectively. MiR‐139‐5p was down‐regulated, whereas *HOXA9* was up‐regulated in OSCC tissues and cells. The proliferation, invasion and migration ability of SAS and CAL‐27 cells in miR‐139‐5p mimics group were significantly weaker than those in the control group and the miR‐NC group (*P *<* *0.01). MiR‐139‐5p can negatively regulate *HOXA9*. The proliferation, invasion and migration of SAS and CAL‐27 cells in the miR‐139‐5p mimics + pEGFP‐*HOXA9* group were not significantly different from those in the blank control and negative control groups (*P *>* *0.05). Our results indicated that miR‐139‐5p could directly inhibit *HOXA9*, which might be a potential mechanism in inhibiting the proliferation, invasiveness and migration of OSCC cells.

## Introduction

Oral cancer originates from the epithelial cells of the oral cavity, which is widely known as one of the most frequently developed malignant tumours among head and neck carcinomas 1. Pathologically, over 90% of all patients suffering from oral cancer are diagnosed as OSCC with well or moderately differentiation 2. When it comes to the treatment of OSCC, we often give priority to surgery with adjuvant radiotherapy or systemic chemotherapy based on the pathological type, the disease stage, patients’ physical state and other correlated factors 3. In spite of advanced therapy, the 5‐year survival rate of OSCC remains unsatisfactory and even lower than 50% when diagnosed during the middle or late stage of the disease 4. These following cancers are malignant, and have a tendency to attack locally, they are relative with a high risk of recurrence 5 **.**The poor prognosis of OSCC can be attributed to the comprehensive factors including the late diagnosis, radiation resistance, recurrence at the primary site and frequent distant metastasis after treatment 6, 7. The mechanism that is responsible for tumour cells migration and invasion is complicated, which remains unknown. Therefore, more efforts need to make in accounting the potential molecular mechanism for OSCC tumorigenesis.

MicroRNAs (miRNAs) are a large family of non‐coding endogenous RNA molecules, consisting of 18‐22 nucleotides 8. MiRNAs execute functions in RNA silencing by binding to the 3′‐untranslated region (UTR) of the target messenger RNAs (mRNAs) 9. In the past decades, emerging evidence has demonstrated that miRNAs play important roles in diverse biological processes including cell proliferation, metastasis, survival, autophagy and differentiation 10, 11. A large sum of miRNAs has been described as cancer genes and their deregulations have been measured in a series of tumours, including OSCC 12. Furthermore, recent studies have revealed that miRNAs serve as either onco‐miRNAs or tumour suppressors during cancer progression and tumorigenesis 13, 14. Previous research has demonstrated that a variety of miRNAs that are abnormally expressed in OSCC, such as miR‐301b, miR‐96, miR‐194, miR‐221 and miR‐139‐5p 15, 16, 17, 18, 19. MiR‐139‐5p has been reported to be down‐regulated in various carcinomas, such as bladder cancer, colorectal cancer, prostate cancer and breast cancer 20, 21, 22, 23. Besides, it was documented to participate in regulating cell proliferation and invasion of human non‐small cell lung cancer 24, oesophageal squamous cell carcinoma 25, hepatocellular carcinoma 26, glioblastoma multiforme 19 and so on. However, studies about the regulatory role of miR‐135‐5p for OSCC cells remained limited; therefore, the clinical value and prognostic potential of miR‐139‐5p for OSCC still needed to be further investigated. The targeted relationship of miR‐139‐5p and HOXA9 has been confirmed in other disorders, such as acute myeloid leukaemia 27, and HOXA9 has been indicated to be involved in development of OSCC 28. Moreover, *HOXA9* also participated in the development of other carcinomas. For instance, Li *et al*. detected that *HOXA9* had essential oncogenic effect in leukaemia 29. Besides, Liu *et al*. found that the expression of *HOXA9* could be enhanced by miR‐196b in chronic myeloid leukemogenesis 30. Mammalian HOX genes are organized in four clusters (HOXA, HOXB, HOXC and HOXD), each containing nine to 11 genes 31. *HOXA9*, 180‐nucleotide in length, is a member of mammalian HOX family 32. *HOXA9* is required to maintain the development of proper limb, skeletal, mammary gland, urogenital tract and kidney 33. *HOXA9* also plays a role in normal myeloid, erythroid and lymphoid haematopoiesis 34. The overexpression of *HOXA9* remarkably expands haematopoietic stem cells, suggesting its function in early haematopoiesis and thus, it might affect the angiogenesis during the tumorigenesis process 32.

Here, we quantified the expression of miR‐139‐5p and *HOXA9* mRNA in 40 OSCC tissue samples by qRT‐PCR. MiR‐139‐5p was significantly down‐regulated in both tissues and cells, whereas *HOXA9* was completely opposite. Dual luciferase reporter assay verified that miR‐139‐5p directly targeted *HOXA9*. Furthermore, cellular assays were, respectively, performed *in vitro* to detect the role that miR‐139‐5p plays in a series of cell activities. In all, we found that miR‐139‐5p severed as an anti‐oncogenic role in OSCC and suppressed cell proliferation, invasion and migration through inhibiting *HOXA9*.

## Materials and methods

### Patients and samples

Forty cases of OSCC tissues and corresponding adjacent tissues were obtained from OSCC patients who underwent OSCC resection without receiving preoperative chemotherapy or radiation at Henan Provincial People's Hospital from January 2014 to January 2016. Among the patients, there were 24 men and 16 women, and the average age of the patients was around 49.8 ± 5.3 years old. The OSCC tissues were all pathologically identified as OSCC, and the numbers of high‐differentiated, medium‐differentiated and poor‐differentiated cases were, respectively, 13, 8 and 19. According to the tumour node metastasis (TNM) staging, the included OSCC tissues were classified as stage I (*n* = 5), stage II (*n* = 10), stage III (*n* = 15) and stage IV (*n* = 10). Tissue samples were immediately frozen in liquid nitrogen and stored at −80°C until analysis. The study was approved by the ethics committee of Henan Provincial People's Hospital. All patients included in this study have signed consent forms.

### Cell culture

The normal oral keratinocyte NOK cell line and human OSCC cell lines SAS, TCA8113, KON were supplied by Henan Provincial People's Hospital cell centre and CAL‐27 cell line was purchased from ATCC. All cells were all cultured in 10% foetal bovine serum (FBS) dulbecco's modified eagle medium (DMEM) at 37°C in a humidified atmosphere with 5% CO_2_.

### Quantitative real‐time PCR (qRT‐PCR)

Total RNA was extracted from the tumour tissues and adjacent tissues or cells using Trizol RNA extraction kit. Then, we reverse‐transcribed the RNAs to cDNAs using RT Kit (Fermentas, Waltham, MA, USA), and cDNAs were amplified using PCR Kit (Invitrogen, Carlsbad, CA, USA). We listed the used primers in Table 1. The mRNA expressions were the relative expression compared with U6 and GAPDH expressions in the same samples. U6 served as the internal control for miR‐139‐5p, and GAPDH served as the internal control for HOXA9. And the $2^{-▵▵c_{t}}$ method was used to calculate their relative expressions.

**Table 1:** The sequences of primers used in qRT‐PCR

Primer	Sequence (5′–3′)
miR‐139‐5p (F)	5′‐ GCCTCTACAGTGCACGTGTCTC ‐3′
miR‐139‐5p (R)	5′‐ CGCTGTTCTCATCTGTCTCGC ‐3′
U6 (F)	5′ ‐ CTCGCTTCGGCAGCACA ‐3′
U6 (R)	5′‐ AACGCTTCACGAATTTGCGT‐3′
HOXA9 (F)	5′‐ CCACGCTTGACACTCACACT ‐3′
HOXA9 (R)	5′‐ CAGTTCCAGGGTCTGGTGTT ‐3′
GAPDH (F)	5′‐ ACAACTTTGGTATCGTGGAAGG ‐3′
GAPDH (R)	5′‐ GCCATCACGCCACAGTTTC ‐3′

### Cell transfection

Cells were seeded into six‐well plates at 30% concentration in 2 ml of supplemented DMEM and cultured for 24 hrs. Then 50 nM of miR‐139‐5p mimics (miR‐mimics), mimics control (miR‐NC), pEGFP‐N1‐3FLAG‐*HOXA9*‐GFP plasmids (pEGFP‐*HOXA9*), empty pEGFP plasmids (pEGFP‐NC) and miR‐139‐5p mimics + pEGFP‐*HOXA9* (50 nM; Shanghai GenePharma Co. Ltd., Shanghai, China) were, respectively, transfected to SAS and CAL‐27 cells using Lipofectamine 2000 according to the instructions of manufacturer (Invitrogen).Cells in the control group were without transfection. After post‐transfection for 48 hrs, cells were harvested.

### MTT assay

Cells of each group were plated into 96‐well plates at 5 × 10^3^ cells/well. Then after incubation of the cells for 24, 48 and 72 hrs, respectively, we added 20 μl MTT solutions (5 mg/ml) to each well. After incubated for 4 hrs, we added 150 μl DMSO to each well in order to promote the dissolution of crystals. Cell viability was detected at 0, 24, 48 and 72 hrs, and the OD was measured on a microplate reader at 490 nm.

### Colony formation assay

Cells of each group were plated into six‐well plates at a concentration of 500 cells/well after transfection for 24 hrs. After the cells adhered to the wall, 0.1% DMSO was applied to act on the cells for 10 days. After being washed with PBS, the cells were fixed by 4% paraformaldehyde, and stained with 5% crystal violet. Then the numbers of colonies were recorded.

### Transwell assay

Cells of each group were plated into the upper Transwell chamber at a concentration of 1 × 10^5^ cells/ml, and 600 μl 10% FBS DMEM was added to the lower chamber. After incubation for 24 hrs, the membrane was fixed by 70% ethanol and stained with 0.1% crystal violet. Finally, the number of cells that passed across the membrane was counted in six randomly selected fields under the microscope.

### Wound healing assay

Cells of each group were plated in 35 mm^2^ culture dish at 8 × 10^5^ cells/dish. When the cells grew to a degree that 90% cells were merged, lines were drawn at the bottom of dish with a mark as markers, and scratched at the cell layers of each group with a 200 μl sterile pipette. After cells were incubated for 0, 24 or 48 hrs later, photographs were taken, respectively. The intersection was considered as the observation point, and the open wound rate was measured using a microscope.

### Luciferase reporter assay

The wild‐type and mutated sequences of *HOXA9* 3 ‘UTR were cloned into pGL3‐M (Promega, Madison, WI, USA) using Xba I and Pst I restriction sites. PGL3‐*HOXA9*‐3′UTR‐wt and pGL3‐*HOXA9*‐3 ‘UTR‐mut recombinant vectors were generated and verified by sequencing. MiR‐139‐5p mimics or mimics control was cotransfected into cells with pGL3‐*HOXA9*‐3 ‘UTR‐wt or pGL3‐*HOXA9*‐3 ‘UTR‐mut. After the transfection of 48 hrs, luciferase activity of cells was detected using dual luciferase reporter gene system.

### Western blotting

After culturing the transfected cells for 48 hrs, the total protein was extracted. The electrophoretic separation with 10% dodecyl sulphate, sodium salt‐polyacrylamide gel electrophoresis (SDS‐PAGE) was conducted, and each lane was loaded with 50 μg of total proteins. The total proteins were then transferred to polyvinylidene fluoride (PVDF) membrane which was subsequently blocked for 1.5 hrs using 5% skim milk. Primary antibodies of HOXA9 and GAPDH (Abcam, Cambridge, MA, USA) diluted at 1:1000 were added onto the membrane and stained overnight at 4°C. After the membrane was washed three times using TBST, goat anti‐rabbit HRP horseradish peroxidase‐labelled antibodies (1:1000) were added and incubated. electro‐chemi‐luminescence (ECL) was used to develop the film.

### Data analysis and statistical methods

Receiver operating characteristic (ROC) curve analysis was performed using SPSS 15.0 (IBM, Armonk, New York, USA) to calculated for evaluated the predictive ability of miR‐139‐5p to distinguish OSCC patients from normal patients. Statistical analyses were conducted using SPSS 21.0 and GraphPad Prism 6.0 (GraphPad Software Inc., San Diego, CA). Measurement data were recorded as mean ± S.D. The comparison between two groups was analysed using *t‐*test, whereas one‐way anova test was used for the analyses among more than two groups, followed by *post hoc* Bonferroni's test. *P *<* *0.05 was statistically significant.

## Results

### MiR‐139‐5p was under‐expressed in OSCC tissues and diverse OSCC cells

**Figure 1:**
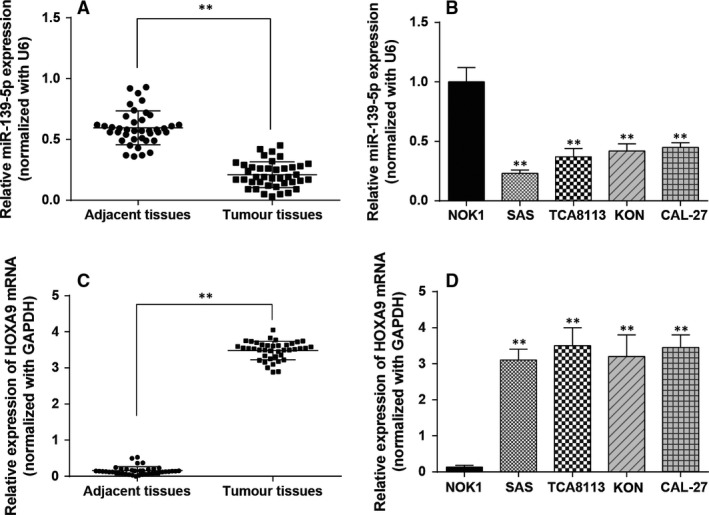
Expression levels of miR‐139‐5p and *HOXA9*. (**A**) MiR‐139‐5p was detected with qRT‐PCR in OSCC tissues and adjacent non‐tumorous tissues. (**B**) MiR‐139‐5p was measured by qRT‐PCR in OSCC cells *versus* normal cells. (**C**) The *HOXA9* mRNA expression was measured by qRT‐PCR in OSCC tissues and adjacent non‐tumorous tissues. (**D**) The *HOXA9* mRNA expression was detected with RT‐PCR in OSCC cells *versus* normal cells. Data are expressed as mean ± S.D. **P <* *0.01, compared with the adjacent tissues or the normal cell line NOK1. #*P *<* *0.05, compared with the SAS cell line. OSCC: oral squamous cell carcinoma.

The miR‐139‐5p level in OSCC tissues and adjacent tissues was measured by qRT‐PCR, which indicated that miR‐139‐5p in OSCC tissues was remarkably decreased compared to adjacent tissues (*P *<* *0.01, Fig. 1A). MiR‐139‐5p expression was also remarkably down‐regulated in four OSCC cell lines SAS, TCA8113, KON and CAL‐27 compared with the normal cell line (*P *<* *0.01, Fig. 1B). The succeeding experiments were conducted in SAS cell line in which miR‐139‐5p expression was the lowest. The expression of *HOXA9* mRNA in OSCC tissue samples and adjacent tissues was compared by qRT‐PCR and results demonstrated a remarkable increasing of *HOXA9* mRNA in OSCC tissues compared to adjacent tissues (*P *<* *0.01, Fig. 1C). *HOXA9* mRNA expression level was remarkably up‐regulated in four OSCC cells SAS, TCA8113, KON and CAL‐27 compared with the normal cell line (*P *<* *0.01, Fig. 1D). Further analysis in Table 2 showed that miR‐139‐5p expression was strongly associated with tumour size and clinical stage, whereas *HOXA9* level was correlated to pathological differentiation according to Fisher's exact test (*n* = 40). Probably limited by small sample size, no significant association among miR‐139‐5p, HOXA9, age, gender and LN metastasis was obtained.

**Table 2:** The baseline characteristics of OSCC patients included (*n* = 40)

Characteristic	Case.	miR‐139‐5p expression		*P* value	HOXA9 expression		*P* value
		Low	High		Low	High	
Age							
≤50	17	7	10	0.5231	11	6	0.2003
>50	23	13	10		9	14	
Gender							
Male	24	14	10	0.3332	13	11	0.7475
Female	16	6	10		7	9	
Tumour size							
≤4 cm	26	9	17	**0.0187**	15	11	0.3203
>4 cm	14	11	3		5	9	
Differentiation							
High/Moderate	21	8	13	0.2049	15	6	**0.0104**
Poor	19	12	7		5	14	
Clinical stage							
I–II	15	4	11	**0.0484**	7	8	1
III–IV	25	16	9		13	12	
LN metastasis							
No	23	13	10	0.5231	10	13	0.5231
Yes	17	7	10		10	7	
		20	20		20	20	

LN: Lymph node; HOXA9: homeobox A9.

*P* value was obtained with Fisher's exact test.

*P *<* *0.05 represents significant differences.

Bold type indicates statistically significant difference.

### MiR‐139‐5p inhibited the viability, proliferation, invasion and migration of SAS and CAL‐27 cell lines

**Figure 2:**
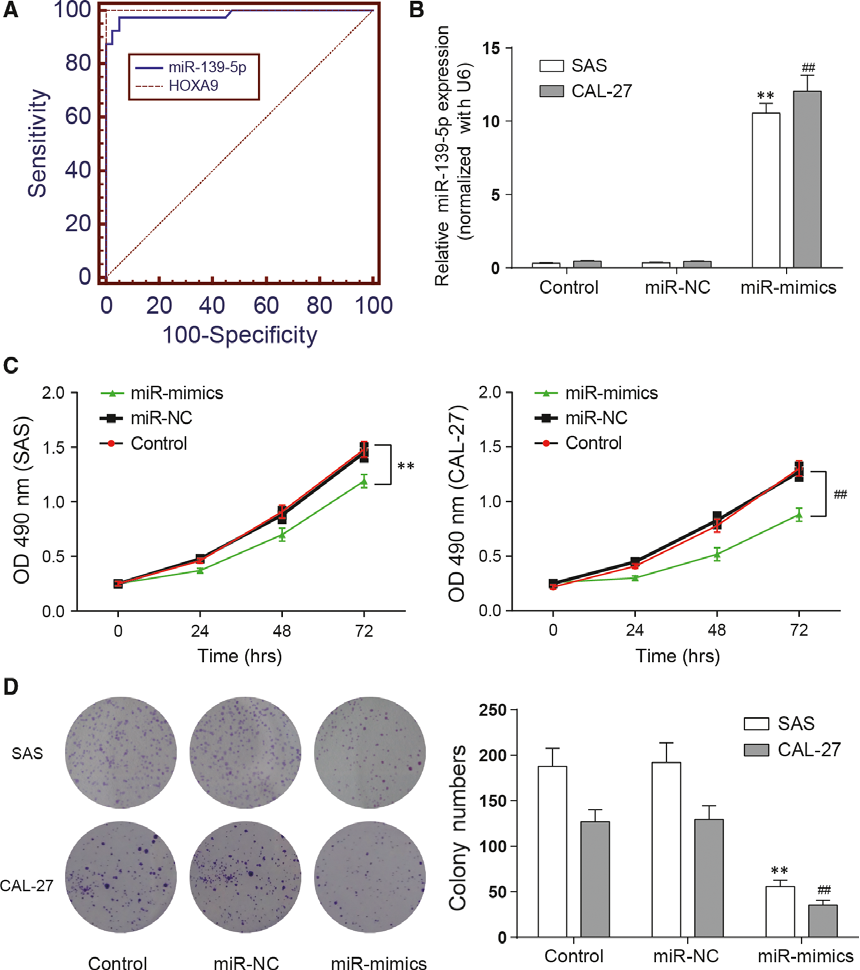
MiR‐139‐5p suppresses proliferation of OSCC cells. (**A**) Receiver operating curves(ROC) comparing miR‐139‐5p and *HOXA9* level in OSCC tissues and adjacent tissues. (**B**) MiR‐139‐5p was detected with qRT‐PCR in each group including control, miR‐negative control (NC) and miR‐mimics of SAS and CAL‐27 cell lines. (**C**) Cell viability was analysed by MTT assay. (**D**) Cell proliferation was analysed by colony formation assay. Data are expressed as mean ± S.D. **P <* *0.01 compared with control in SAS cell line, ## *P *<* *0.01 compared with control in CAL‐27 cell line. Control: control group, cells were without transfection. MiR‐NC: miR‐NC group, cells transfected with mimics control. MiR‐mimics: miR‐mimics group, cells transfected with miR‐139‐5p mimics.

Firstly, we generated ROC curves and found that miR‐139‐5p exhibited AUC of 0.985, *HOXA9* exhibited AUC of 1 (Fig. 2A), indicating sufficient power to distinguish OSCC tissues from adjacent tissues. Then, results of qRT‐PCR suggested that the miR‐139‐5p expression in miR‐139‐5p mimics group is significantly higher than that in control and miR‐NC groups in both cell lines(**P <* *0.01 compared with control in SAS cell line, ## *P *<* *0.01 compared with control in CAL‐27 cell line, Fig. 2B). Next, results of MTT assay showed that cells of miR‐139‐5p mimics group had significantly lower viability than control and miR‐NC groups (**P <* *0.01 compared with control in SAS cell line, ## *P *<* *0.01 compared with control in CAL‐27 cell line, Fig. 2C), suggesting that miR‐139‐5p inhibited the viability of SAS and CAL‐27 cell lines Colony formation assay was used to detect the colony forming ability. Similar to the results of MTT assay, as shown in Figure 2D, both cell lines in miR‐139‐5p mimics group had fewer colony formations than control groups (**P <* *0.01 compared with control in SAS cell line, ## *P *<* *0.01 compared with control in CAL‐27 cell line), indicating that miR‐139‐5p could inhibit SAS and CAL‐27 cells proliferation.

**Figure 3:**
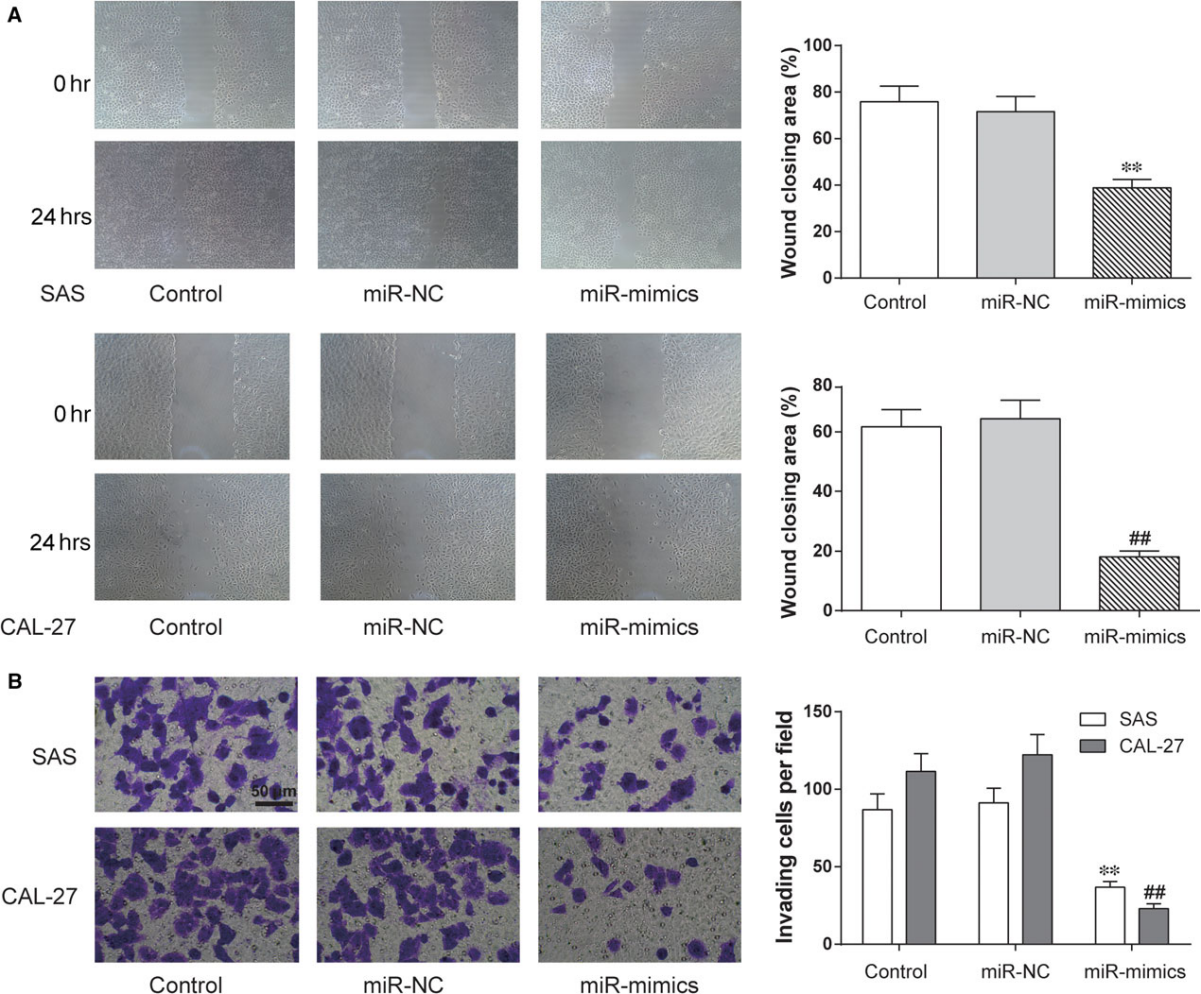
MiR‐139‐5p suppresses migration and invasion of OSCC cells. (**A**) After replenishing miR‐139‐5p expression in SAS and CAL‐27 cells, cell migration was assessed by wound healing assay. (**B**) Cell invasion ability was analysed by Transwell assay. **P <* *0.01 compared with control in SAS cell line, ## *P *<* *0.01 compared with control in CAL‐27 cell line. Control: control group, cells were without transfection. MiR‐NC: miR‐NC group, cells transfected with mimics control. MiR‐mimics: miR‐mimics group, cells transfected with miR‐139‐5p mimics.

Wound healing assay was used to assess the migration of SAS and CAL‐27 cells. The results suggested that open wound rate of cells in the control and miR‐NC groups surpassed that in the miR‐139‐5p mimics group (**P <* *0.01 compared with control in SAS cell line, ## *P *<* *0.01 compared with control in CAL‐27 cell line, Fig. 3A). The results revealed that miR‐139‐5p inhibited the migration of SAS and CAL‐27 cells. And the invasion of SAS and CAL‐27 cells was measured by Transwell assay. The results of transwell assay indicated that cells in the miR‐139‐5p mimics group had weaker invasiveness than cells in the control groups (**P <* *0.01 compared with control in SAS cell line, ## *P *<* *0.01 compared with control in CAL‐27 cell line, Fig. 3B), showing that miR‐139‐5p inhibited the invasion of SAS and CAL‐27 cells.

### *HOXA9* is the target of miR‐139‐5p

**Figure 4:**
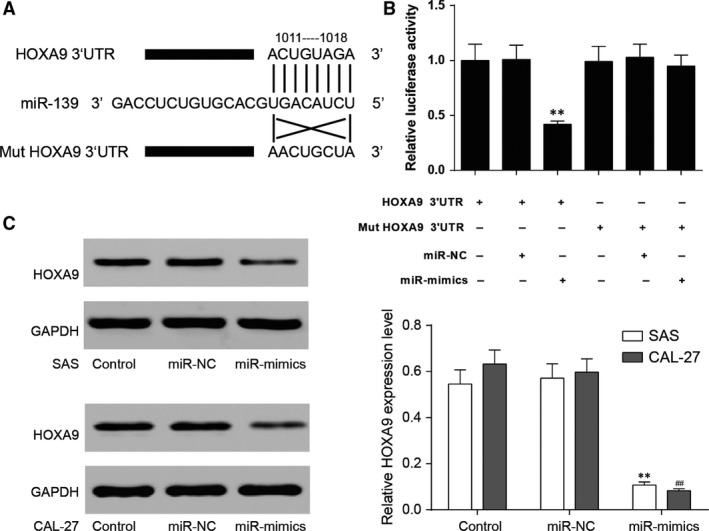
MiR‐139‐5p targeted *HOXA9*. (**A**) The predicted binding site of miR‐139‐5p was in the 3 ‘UTR of *HOXA9*. (**B**) HEK293 cells were transfected with either miR‐139‐5p mimics or miR‐ negative control (NC) and *HOXA9*‐wt or *HOXA9*‐mut. The luciferase activity was analysed after 48 hrs. (**C**) The protein expression of *HOXA9* in SAS and CAL‐27 cell lines was detected by Western blot. GAPDH was served as an internal control. Data are expressed as mean ± S.D. **P <* *0.01 compared with control in SAS cell line, ## *P *<* *0.01 compared with control in CAL‐27 cell line. Control: control group, cells were without transfection. MiR‐NC: miR‐NC group, cells transfected with mimics control. MiR‐mimics: miR‐mimics group, cells transfected with miR‐139‐5p mimics.

*HOXA9* was predicted to be a target of miR‐139‐5p with the possible binding sequence ACUGUAGA in *HOXA9* 3′UTR by the online database TargetScan Human 7.0 (Fig. 4A). The luciferase activity of *HOXA9*‐wt 3′UTR + miR‐139‐5p mimics decreased significantly (*P *<* *0.01, Fig. 4B), while that of *HOXA9*‐mut 3′UTR + miR‐139‐5p mimics was not significantly different from the control groups. It suggested that miR‐139‐5p can directly inhibit *HOXA9*.

The suppression effect of miR‐139‐5p on HOXA9 was evaluated at protein levels by Western blot. HOXA9 protein level was down‐regulated in SAS and CAL‐27 cells transfected with miR‐139‐5p mimics in comparison with the control groups, further verifying the inhibition of miR‐139‐5p on HOXA9 (**P <* *0.01 compared with control in SAS cell line, ## *P *<* *0.01 compared with control in CAL‐27 cell line, Fig. 4C).

### MiR‐139‐5p inhibited the viability of SAS and CAL‐27 cells by suppressing *HOXA9*

**Figure 5:**
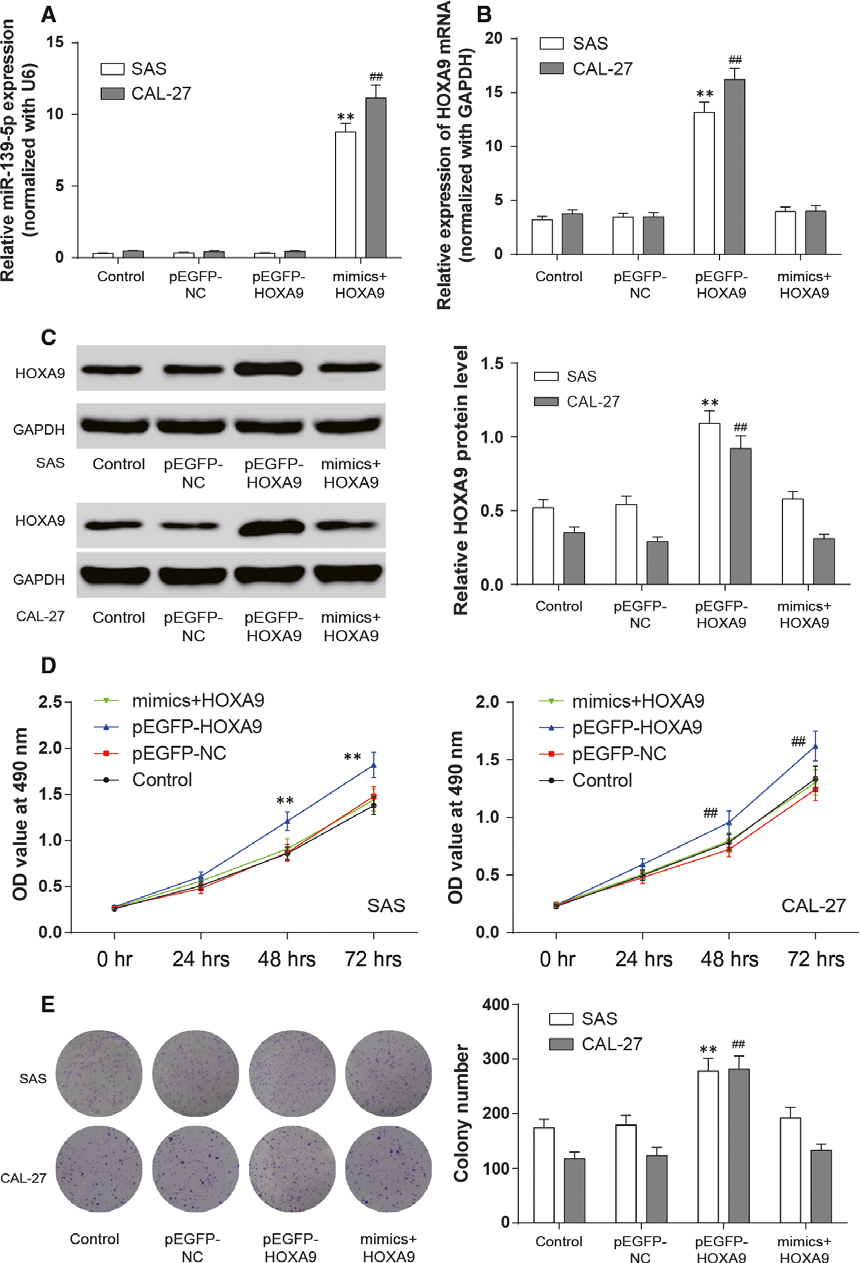
MiR‐139‐5p suppressed the proliferation of OSCC cells by targeting *HOXA9*. (**A** and **B**) Expression of miR‐139‐5p and *HOXA9* mRNA was detected with qRT‐PCR in each group. (**C**) The protein level of HOXA9 in SAS and CAL‐27 cell lines was assessed by Western blot. GAPDH was used as an internal control. (**D**) Cell viability was analysed by MTT assay. (**E**) Cell proliferation was detected through colony formation assay. Data are expressed as mean ± S.D. **P <* *0.01 compared with control in SAS cell line, ##*P *<* *0.01 compared with control in CAL‐27 cell line. Control: control group, cells were without transfection. pEGFP‐NC: pEGFP‐NC group, cells transfected with empty pEGFP plasmids. pEGFP‐*HOXA9*: pEGFP‐*HOXA9* group, cells transfected with pEGFP‐N1‐3FLAG‐*HOXA9*‐GFP plasmids. MiR‐139‐5p mimics + pEGFP‐*HOXA9*: miR‐139‐5p mimics + pEGFP‐*HOXA9* group, cells cotransfected with miR‐139‐5p mimics and pEGFP‐*HOXA9*.

Firstly, the results of qRT‐PCR demonstrated that the miR‐139‐5p expression in miR‐139‐5p mimics + pEGFP‐*HOXA9* group was remarkable higher than that of other groups (**P <* *0.01 compared with control in SAS cell line, ## *P *<* *0.01 compared with control in CAL‐27 cell line, Fig. 5A). It turned out that the expression of miR‐139‐5p was not affected by the overexpression of *HOXA9*. Then, in Figure 5B and C, HOXA9 mRNA expression and protein level were significantly up‐regulated in pEGFP‐*HOXA9* group compared with the pEGFP‐NC1 group (**P <* *0.01 compared with control in SAS cell line, ## *P *<* *0.01 compared with control in CAL‐27 cell line), while HOXA9 protein level was almost identical in miR‐139‐5p mimics + pEGFP‐*HOXA9* and pEGFP‐NC2 groups. The results showed that miR‐139‐5p inhibited the expression of *HOXA9*.

In MTT assay, the overexpression of *HOXA9* (pEGFP‐*HOXA9* group) significantly increased cell viability, whereas the viability of SAS and CAL‐27 cells in miR‐139‐5p mimics + pEGFP‐*HOXA9* group were not significantly different from that in corresponding controls (*P *> 0.05, Fig. 5D). These findings indicated that miR‐139‐5p could eliminate the increased cell viability of OSCC cells induced by the overexpression of *HOXA9*.

### MiR‐139‐5p inhibited the proliferation, invasion and migration of SAS and CAL‐27 cells by suppressing *HOXA9*

The cell proliferation of miR‐139‐5p on *HOXA9* was assessed by colony formation assay. Compared with the control and pEGFP‐NC groups, the pEGFP‐*HOXA9* group formed more colonies while the colony formation of SAS and CAL‐27 cells in miR‐139‐5p mimics + pEGFP‐*HOXA9* group were not different from that in corresponding control groups (**P <* *0.01 compared with control in SAS cell line, ## *P *<* *0.01 compared with control in CAL‐27 cell line, Fig. 5E).

**Figure 6:**
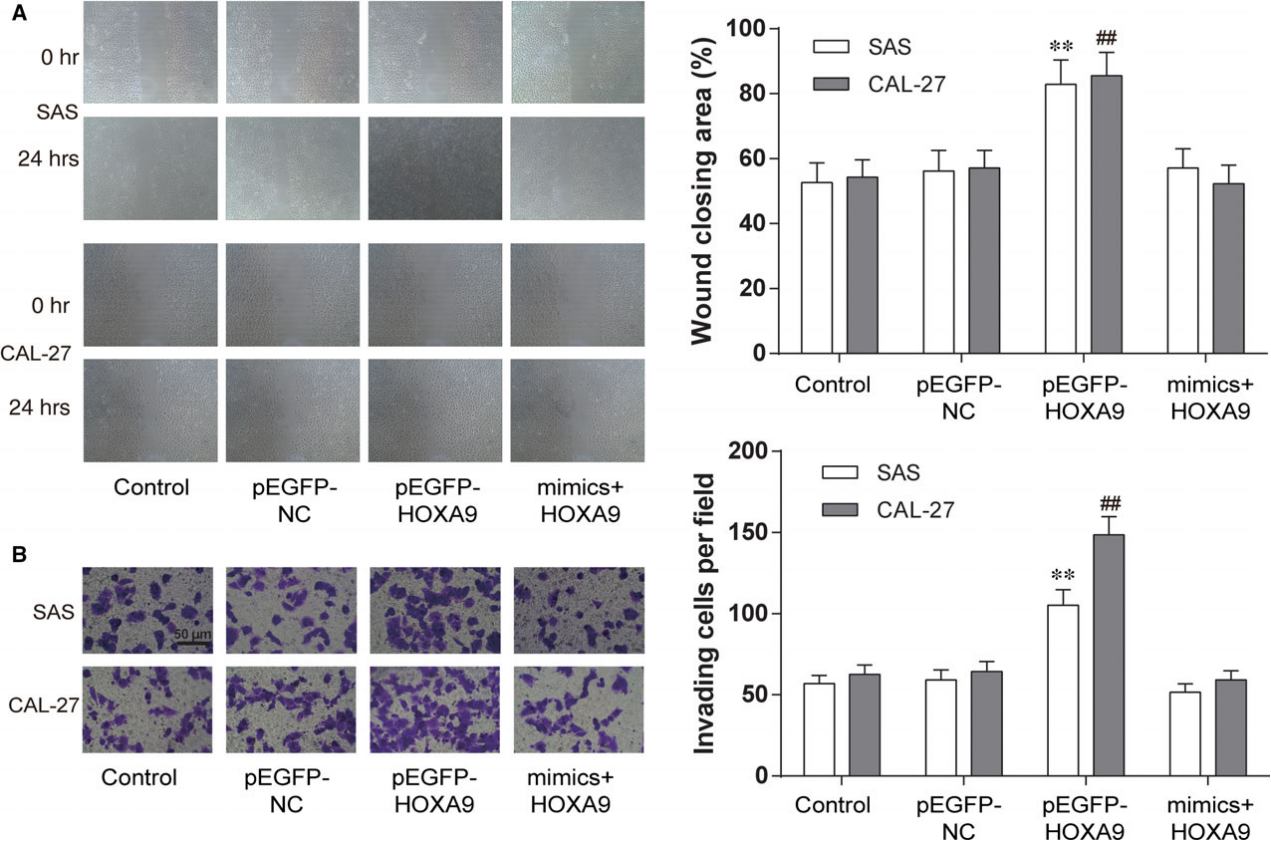
MiR‐139‐5p attenuated migration and invasion of OSCC cells by targeting *HOXA9*. (**A**) At 48 hrs after transfection, cell migration was assessed by wound healing assay. (**B**) Cell invasion was analysed by Transwell assay. Data are expressed as mean ± S.D. **P <* *0.01 compared with control in SAS cell line, ## *P *<* *0.01 compared with control in CAL‐27 cell line. Control: control group, cells were without transfection. pEGFP‐NC: pEGFP‐NC group, cells transfected with empty pEGFP plasmids. pEGFP‐*HOXA9*: pEGFP‐*HOXA9* group, cells transfected with pEGFP‐N1‐3FLAG‐*HOXA9*‐GFP plasmids. MiR‐139‐5p mimics + pEGFP‐*HOXA9*: miR‐139‐5p mimics + pEGFP‐*HOXA9* group, cells cotransfected with miR‐139‐5p mimics and pEGFP‐*HOXA9*.

The cell migration of SAS and CAL‐27 cells was detected by wound healing assay. As shown in Figure 6A, the open wound rate of cells in the pEGFP‐*HOXA9* group was higher than that in the control groups. No significant difference in the open wound rate was observed among the miR‐139‐5p mimics + pEGFP‐*HOXA9* group, the the control group and the pEGFP‐NC group (**P <* *0.01 compared with control in SAS cell line, ## *P *<* *0.01 compared with control in CAL‐27 cell line).

Then Transwell assay was conducted to confirm the suppression effect of miR‐139‐5p on HOXA9 in cell invasion. The pEGFP‐HOXA9 group had stronger invasiveness in comparison with the control group and the pEGFP‐NC group. There was no remarkable difference in the invasiveness of SAS and CAL‐27 cells among the miR‐139‐5p mimics + pEGFP‐HOXA9 group, the control group and the pEGFP‐NC group (**P < 0.01 compared with control in SAS cell line, ## *P* < 0.01 compared with control in CAL‐27 cell line, Fig. 6B).

## Discussion

More attention is paying to the explosion of potential molecular mechanism responsible for OSCC due to its increased incidence in both developed and developing countries 35, 36, 37, 38. Among previous studies, most abnormally expressed miRNAs were discovered in the OSCC. For instance, Stuart Hunt *et al*. found that miR‐124 suppressed OSCC motility by targeting ITGB1 39. Shiah *et al*. demonstrated that down‐regulated miR‐329 and miR‐410 promoted the proliferation and invasion of OSCC cells by targeting wnt‐7b 40. Wang *et al*. detected that miR‐433 inhibits OSCC cell growth and metastasis by targeting HDAC6 41. Of note, a number of target genes can be regulated by a single miRNA instantly. For example, it has been reported that miR‐139‐5p suppresses the non‐small cell lung cancer cell proliferation and induces apoptosis *via* inhibiting c‐Met 42. Besides, miR‐139‐5p is able to repress the activation of AMFR and NOTCH1 in colorectal cancer 43. Also, miR‐139‐5p functions as a master regulator of glioblastoma metastasis through targeting ZEB1 and ZEB2 19. Furthermore, the increased expression level of miR‐139‐5p can lead to suppressed cell viability and proliferation in various cancers through cell cycle arrest, which induces more cells arrested at G0/G1 or S phase 44, 45. Besides, cell cyclin involving E1, D1, MMP2 and MMP9 is significantly changed in cell transfected with miR‐139‐5p mimics 25, 46. Furthermore, a decrease in the apoptotic protein such as Bax 47, 48 and an increase in the anti‐apoptotic protein including Bcl‐2 as well as Mcl‐1 47, 49, regulated by miR‐139‐5p, also participates in the tumour process. In the present study, miR‐139‐5p was found down‐regulated in the OSCC sample tissues, and its up‐regulation suppressed the viability, proliferation, migration and invasiveness of OSCC cells. Notably, emerging evidence has highlighted that miR‐139‐5p served as a tumour suppressor in various carcinomas, including bladder cancer, colorectal cancer, prostate cancer and breast cancer 20, 21, 22, 23. Besides, miR‐139‐5p has also been confirmed as a prognostic element for progression of breast cancer 50. Therefore, the suppressor role of miR‐139‐5p in OSCC proposed in our study was convincing, and we should explore it further.

To better understand the precise molecular mechanism that is responsible for the antitumour effects of miR‐139‐5p in OSCC, we searched potential target genes of miR‐139‐5p using TargetScan (http://www.targetscan.org). *HOXA9* was predicted to harbour one highly conservative miR‐139‐5p binding sites in the sequence of 3′UTR. With the use of dual luciferase reporter assays, *HOXA9* was eventually identified as a direct target for miR‐139‐5p. The increase in miR‐139‐5p expression was accompanied by the down‐regulated *HOXA9* expression. QRT‐PCR results revealed that *HOXA9* was up‐regulated in SAS cells, which was inconsistent with some of the previous studies. Ruiz *et al*. found that *HOXA9* was under‐expressed in cervical cancer cells and its restoration decreased the proliferation, migration and expression of epithelial‐to‐mesenchymal transition (EMT) genes 51. Hwang JA *et al*. detected that *HOXA9* inhibits lung cancer cells migration and its hypermethylation is closely linked with the recurrence in non‐small cell lung cancer 52. Miso *et al*. found that the HMGA2‐TET1‐*HOXA9* signalling was coordinately regulated in breast cancer. Both TET1 and *HOXA9* suppressed breast cancer development in nude mice 53. On the contrary, *HOXA9* was believed to simulate ovarian cancer progression by inducing peritoneal macrophages to acquire an M2 tumour‐promoting phenotype 54. Similarly, Ko *et al*. asserted that the *HOXA9* expression in epithelial ovarian cancer cells promised a comfortable environment for tumour growth 55. The work performed by Pojo *et al*. discovered that *HOXA9* promoted human glioblastoma initiation and aggressiveness 56. In terms of the promoter role of *HOXA9* during OSCC development and progression, our results verified that miR‐139‐5p suppressed OSCC cell viability, proliferation, invasion and migration by directly inhibiting *HOXA9*. We also found that the effects of *HOXA9* could be deteriorated by exogenous miR‐139‐5p. But whether *HOXA9* contributed to the alteration of tumour microenvironments need further proved.

Still, there are several limitations in our study. Due to the resource constraint of clinical samples, only 40 pairs of human OSCC tissues and matched adjacent tissues were involved in the present study. Such a small sample size might result in minor errors in the relative expression of miR‐139‐5p. We intended to future research to verify the above conclusions. Besides, the correlated signalling pathway might help explain the molecular mechanism of anti‐oncogenic effect of miR‐139‐5p on OSCC cells. Moreover, we verified the anti‐oncogenic effect of miR‐139‐5p in SAS OSCC cell line *in vitro*, while the other OSCC cell lines as well as the experiments *in vivo* (*i.e*. in established animal models) were lacking. In addition, the role of miR‐139‐5p and *HOXA9* in other mechanisms, such as EMT, also needed to be further investigated, which could provide evidence for discovering novel targets for OSCC. As a result, further researches should pay attention to resolving the above limitations.

## Conclusion

In summary, the present study demonstrated that miR‐139‐5p, functioning as a suppressor in the tumorigenesis process, suppressing OSCC cell mobility by targeting *HOXA9* directly. The relative expression of miR‐139‐5p and *HOXA9* was quantified by qRT‐PCR. Dual luciferase reporter gene assay verified that *HOXA9* was a target for miR‐139‐5p. *In vitro* assays performed with OSCC cells further detected the role of miR‐139‐5p played during tumorigenesis. Combined with previous studies on miR‐139‐5p, we believe that miR‐139‐5p might be a crucial molecular that participates in the OSCC tumorigenesis. Anyway, we hope that the results might provide a therapeutic strategy for the treatment of OSCC.

## Funding

None.

## Author Contributions

Kai Wang and Jun Jin performed the research; Tengxiao Ma and Hongfeng Zhai analysed and interpreted the data; Kai Wang designed the research study and wrote themanuscript.

## Conflict of interest

None.
